# Evaluation and Comparison of Prognostic Multigene Tests in Early‐Stage Breast Cancer: Which Is the Most Effective? A Literature Review Exploring Clinical Utility to Enhance Therapeutic Management in Luminal Patients

**DOI:** 10.1002/mc.23893

**Published:** 2025-02-17

**Authors:** Marianna Rita Brogna, Gerardo Ferrara, Valeria Varone, Angela Montone, MariaRosaria Schiano, Michele DelSesto, Francesca Collina

**Affiliations:** ^1^ Pathology Unit, Istituto Nazionale Tumori‐IRCCS‐Fondazione G. Pascale Naples Italy

**Keywords:** breast cancer, decision‐making process, gene expression profile, multigene prognostic assay, personalized medicine

## Abstract

Breast cancer is the most common malignancy affecting women, marked by significant complexity and heterogeneity. This disease includes multiple subtypes, each with unique biological features and treatment responses. Despite significant advancements in detection and therapy, challenges remain, particularly in managing aggressive forms like triple‐negative breast cancer and overcoming drug resistance. Breast cancer classification and subtype determination are typically performed by immunohistochemistry (IHC) method, which assesses four key markers (ER, PR, HER2, KI67); however, due to the recognized issues with this approach—especially regarding the evaluation of Ki67—there is a risk of misclassification. Patients who may be suitable for chemotherapy could miss possible advantages and only experience needless toxicity as a result of improper treatment decisions. Molecular profiling has improved breast cancer management, enabling the creation of multigene prognostic tests (MPTs) like Oncotype Dx, MammaPrint, Prosigna, Endopredict, and Breast Cancer Index which assess gene expression profiles to more accurately predict recurrence risks. These tools help personalize treatment, identifying patients who can avoid chemotherapy and/or extended endocrine therapy. While many MPTs are available, only Oncotype Dx and MammaPrint have prospective validation, with Prosigna providing additional prognostic insights by incorporating clinical variables. Molecular tests are especially usefull in the “*gray zone*,” which includes tumors measuring between 1 and 3 cm with 0–3 positive lymph nodes and an intermediate proliferation index. However, their clinical utility has not been definitively established, and significant differences exist between them. This article provides an in‐depth analysis of established genomic assays, including testing procedures, clinical validity, utility, diagnostic frameworks, and methodologies. Our comparison aims to improve early breast cancer management by guiding pathologists and oncologists in optimizing the use of genomic assays in clinical practice. By presenting this information, we aim to enhance understanding of the clinical utility and effectiveness of these assays, supporting the development of personalized treatment strategies for early breast cancer patients. Genomic assays offer important insights that can support treatment decisions in early‐stage breast cancer, especially when used alongside other clinical evaluations, predictive tools, and management guidelines. While multiple gene expression profiling tests are available, they classify patients differently and are not interchangeable; therefore, their application should be at the clinician's discretion during the decision‐making process. It is essential that these tests are not the sole factor in determining the best treatment plan: other clinical considerations and patient preferences should also play a significant role in guiding treatment decisions.

## 1 Introduction

Breast cancer is the most prevalent neoplasm affecting women. It is a complex and heterogeneous disease that consists of a variety of entities with distinct biology and clinical behaviors, as well as a variable response to therapy [1].

Advances in treatment and early diagnosis have improved outcomes for breast cancer patients, with an average 5‐year survival rate of 80%. However, brain metastasis worsens the prognosis, reducing median survival to under a year [2].

Research on breast cancer's heterogeneity highlights the importance of personalized treatments based on the tumor's molecular and genetic features. Targeted therapies, such as HER2 inhibitors and hormonal treatments, have greatly improved long‐term survival and reduced recurrence in several patient groups. However, challenges remain, particularly in treating triple‐negative breast cancer and addressing treatment resistance, necessitating further research and innovative approaches [3].

In contemporary clinical practice, prognosis, prediction and treatment selection depend on the assessment of clinical‐pathological factors and the expression of established biomarkers, such as HER2, estrogen receptor (ER), progesterone receptor (PgR), and Ki67 through IHC methodology [4, 5].

As is well documented in the literature, IHC has several limitations that can result in misclassification and incorrect therapy decisions [6]. An accurate treatment plan—including biological treatments, endocrine therapy (ET), and chemotherapy (CT)—is crucial to reducing recurrence and mortality in post‐surgery breast cancer patients. While CT is essential for HER2‐positive and triple‐negative tumors, its additional benefit to hormone therapy in hormone‐positive HER2‐negative tumors is debated, especially for younger patients with small, node‐negative tumors [6, 7].

Multigene prognostic tests evaluate tumor gene expression to provide personalized prognoses and inform treatment decisions in breast cancer. By assessing the risk of distant recurrence for patients undergoing ET, these tests help identify those who can safely forgo additional CT or extended ET. Comprehensive tools for managing early breast cancer (EBC) include genetic tests like Oncotype Dx, MammaPrint, Prosigna, EndoPredict, and Breast Cancer Index (BCI). These tests offer prognostic information and predict the potential benefits of further CT, guiding treatment choices. Each assay analyzes different gene sets, employs distinct methods, and generates risk scores based on specific criteria [8, 9].

The clinical trials validating these assays differed in cohort characteristics, such as menopausal status, nodal involvement, hormone receptor expression, and ET usage. Consequently, the clinical validity and utility of these assays—whether for prognosis or predicting treatment benefit—vary depending on specific contexts, like node‐negative versus node‐positive disease or premenopausal versus postmenopausal patients [10, 11].

The latest American Society of Clinical Oncology ASCO guidelines advise using each genomic assay based on evidence for specific clinical contexts. However, no strongly recommended test exists for certain N1 cases, such as EBC with four or more positive lymph nodes or premenopausal patients with one to three positive nodes. This highlights the need for a more integrated use of genomic assays in routine practice to address these gaps [12, 13]. Most molecular prognostic tests assess gene expression (RNA) from tissue samples collected via surgical excision, cytology, or biopsy. Currently, five validated multigene prognostic tests for breast cancer are available commercially: Oncotype Dx, MammaPrint, Endopredict, Prosigna, and BCI. Oncotype DX and MammaPrint have both prospective and extensive retrospective validation, whereas Prosigna is a second‐generation test that combines clinical variables with gene expression data to classify tumors into molecular subtypes (Luminal A, Luminal B, HER2‐enriched, or basal) and provides a score that indicates the likelihood of distant recurrence [14, 15]. This review aims to (i) provide a comprehensive overview and comparison of clinically validated genomic assays for EBC, (ii) examine supporting evidence across different patient populations, and (iii) evaluate the impact of these assays on healthcare costs and treatment decisions. By consolidating this information, the review seeks to enhance understanding of the clinical utility and effectiveness of genomic assays, supporting the development of personalized treatment strategies for EBC patients.

## 2 Multigen Prognostic Tests (TMP) for the Analysis of Gene Expression Profiles in Endocrine Responsive Breast Cancer

Prognostic tests, as opposed to predictive tests that evaluate the potential response to specific treatments, are designed to identify prognostic biomarkers. These biomarkers reflect the molecular or histopathological traits of the tumor, measured before treatment, and are associated with patient survival or disease progression, regardless of therapy administered [16].

In patients with operable breast cancer, it is essential to consider adjuvant systemic treatment, as polychemotherapy, ET, and biological therapies can significantly reduce the risk of recurrence (ROR) and mortality. This decision should be guided by a comprehensive assessment of prognostic factors, treatment response predictors, expected benefits, comorbidities, and patient preferences. For HER2‐positive (HER2+) and triple‐negative tumors—characterized by a lack of estrogen (ER−) and progesterone (PgR−) receptors—the advantages of CT are well‐established and often critical. However, for hormone‐positive HER2‐negative tumors (ER+/HER2−), the benefit of adding CT to adjuvant hormone therapy is still debated, especially for younger patients with small tumors and no lymph node involvement who are not considered high risk [16, 17].

Traditional methods like IHC, which inform subtype classification and treatment selection, can have intrinsic limitations, leading to potential misclassification. This may result in undertreatment or unnecessary CT, exposing patients to possible toxicity. To address these issues, molecular assays for breast cancer have been developed to support clinicians in decision making process, effectively guiding treatment choices [17, 18].

However, it is crucial to emphasize that these tests do not predict an individual patient's specific response to a particular therapy. This distinction is essential to ensure that treatment plans are grounded in a comprehensive understanding of the tumor's characteristics and the patient's overall clinical context.

## 3 Multigene Prognostic Tests (MPT) in Breast Cancer

Most molecular prognostic tests rely on analyzing gene expression (RNA) from tissue samples taken from the primary tumor post‐surgery or through cytology or biopsy. In the context of tumor cells, this method helps identify distinct gene expression patterns associated with different types of cancer, stages of disease, and responses to treatment. Understanding these patterns allows for a deeper exploration of tumor biology, facilitating the development of more targeted therapies and improving prognostic assessments [19].

In breast cancer, gene expression profiling (GEP) tests are essential for predicting the risk of tumor recurrence. These results allow oncologists to identify high‐risk patients who may benefit from CT alongside adjuvant hormone therapy. Conversely, the tests also help identify patients with a low ROR after 10 years, indicating they may be able to safely avoid adjuvant CT [20].

Several commercially available tests are designed to profile the expression of genes in breast tumor tissue, including: Oncotype DX; Mammaprin; Prosigna (PAM‐50); Endopredict; BCI [20, 21].

These tests can be performed on paraffin‐embedded tissue samples. Although all of them have undergone extensive retrospective evaluations, only Oncotype DX and MammaPrint have substantial prospective validation data that support their clinical utility.

Genomic testing is especially recommended for patients with early‐stage, hormone receptor‐positive, HER2‐negative breast cancer that has been surgically treated, particularly in cases with negative lymph nodes or up to four positive lymph nodes [21].

Genomic tests for breast cancer, such as Oncotype DX and Mammaprint, are not indicated for patients with: (i) Early‐stage breast cancer (EBC) with negative hormone receptors; (ii) HER2‐positive tumors; (iii) triple‐negative tumors (negative for hormone receptors and HER2); (iv) more than four positive lymph nodes.

The primary methods for obtaining GEP include techniques that require analysis on a representative tumor sample containing a sufficient number and percentage of neoplastic cells. Generally (i.e., DNA Microarrays; RNA‐seq; RTq PCR), they involve key steps such as extracting total RNA and converting it into complementary DNA (cDNA); in contrast, the Prosigna test utilizes a direct hybridization method on RNA and incorporates fluorescent molecular barcodes via Nanostring technology, providing a unique approach to GEP [21, 22].

✓. **ONCOTYPE DX**

**Figure 1:**
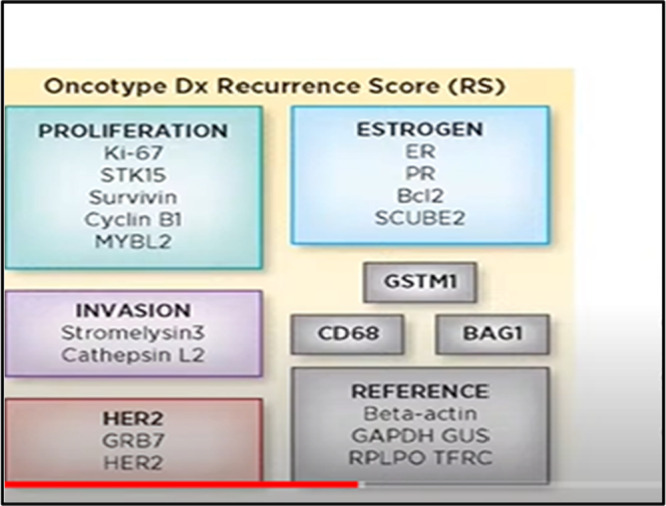
Modules and genes assessed with Oncotype Dx test.

Oncotype DX (Exact Sciences Corp., Madison, WI, USA) is a first‐generation, 21‐gene prognostic and predictive test that has been prospectively validated for both pre‐ and postmenopausal patients with N0 and N1 (1–3 metastatic lymph nodes) HR+/HER2− EBC. The Oncotype DX test is performed centrally and utilizes reverse transcription quantitative PCR (RT‐qPCR) to measure the expression levels of 16 genes associated with breast cancer, normalized to 5 reference genes. The result of the test is a recurrence score (RS), which ranges from 0 to 100, providing an estimate of the patient's risk for distant recurrence [23, 24]. Cut‐off points for the RS from Oncotype DX are used to categorize patients into three risk groups: low risk (RS < 18), intermediate risk (18 ≤ RS ≤ 30), and high risk (RS > 30). Additionally, high RS results (RS > 25) have been shown to predict a potential benefit from adjuvant CT (Figure 1). The RSClin tool, which is available to healthcare professionals via the online platform [https://online.genomichealth.com](https://online.genomichealth.com, accessed on March 20, 2024), integrates the RS with other clinical factors such as tumor grade, size, and patient age. This tool, developed from a meta‐analysis of data from 10,004 women with HR+/HER2− N0 EBC, offers a more personalized risk assessment than clinical‐pathological or genomic data alone [25].

The use of the test is not recommended in HER2‐positive or triple‐negative tumors (TNBCs).

✓. **MAMMAPRINT *(70 GENI)***

**Figure 2:**
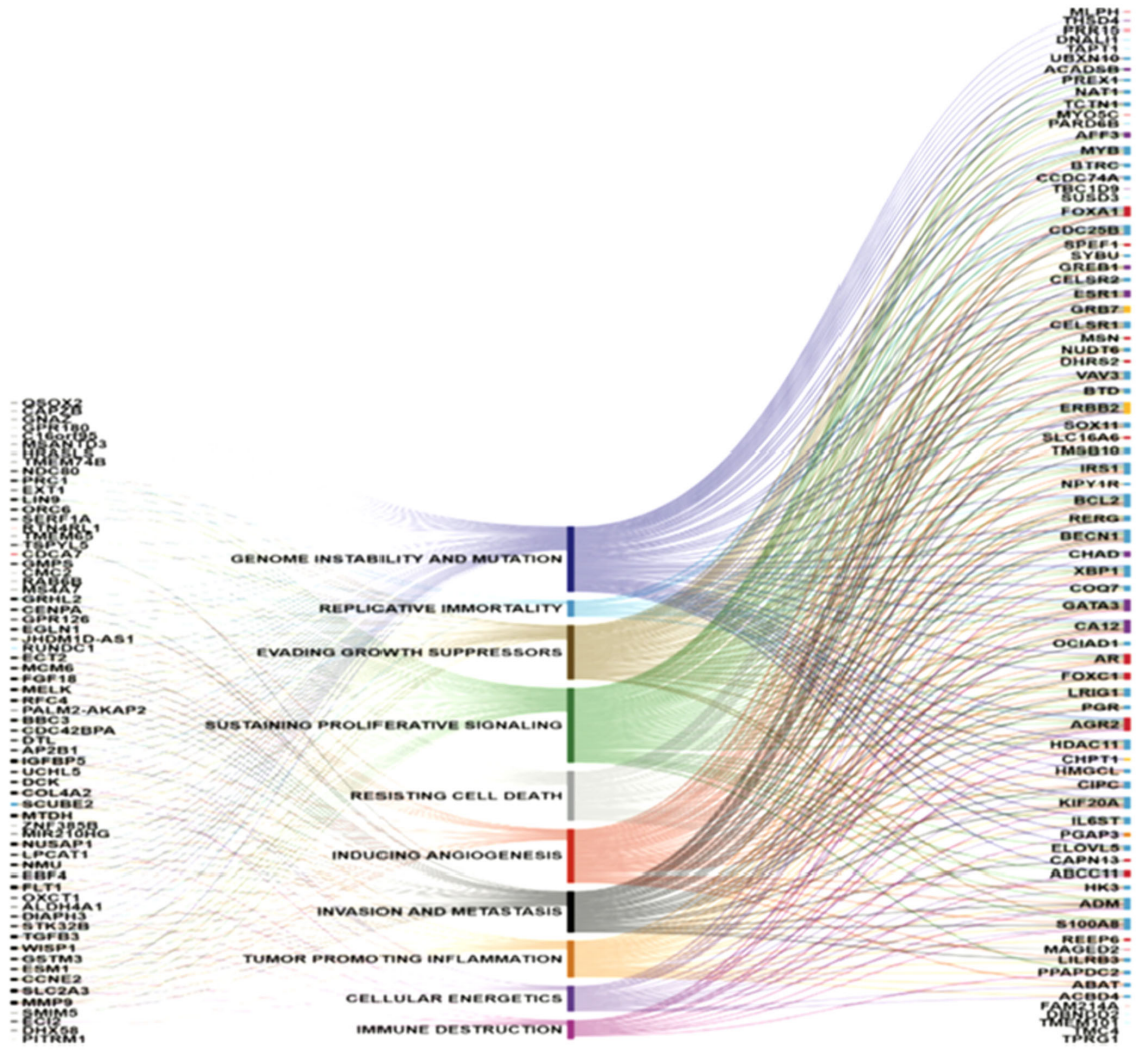
Modules and genes assessed with Mammaprint test.

The ROR in EBC can be evaluated by the first‐generation 70‐gene signature assay MammaPrint (Agendia NV, Amsterdam, The Netherlands). The test was created using information from 20‐year follow‐up EBC patients who had surgery without adjuvant systemic therapy. MammaPrint is a centralized laboratory that uses microarray technology to assess the RNA expression of breast tumor tissue on frozen and formalin‐fixed paraffin‐embedded (FFPE) samples [26]. The combined expression of 70 genes yields an index score ranging from −1 to +1, classifying tumors as having a low (index of 0.001 to 1) or high (index −1 to 0) ROR. Initially, MammaPrint employed a microarray method that could be applied to both frozen and paraffin‐embedded samples, analyzing the expression of 70 genes to create a prognostic molecular signature. This test has since been adapted and validated using next‐generation sequencing (NGS) techniques, resulting in the development of both the MammaPrint and BluePrint tests. The BluePrint component evaluates an additional 80 genes to classify the tumor subtype as luminal, basal, or HER2 (Figure 2) MammaPrint was the first assay to be cleared at the 510(k) level by the US FDA's new in vitro diagnostic multivariate index assay classification and has the CE‐IVD mark [27].

✓. **ENDOPREDICT (12 GENES)**

**Figure 3:**
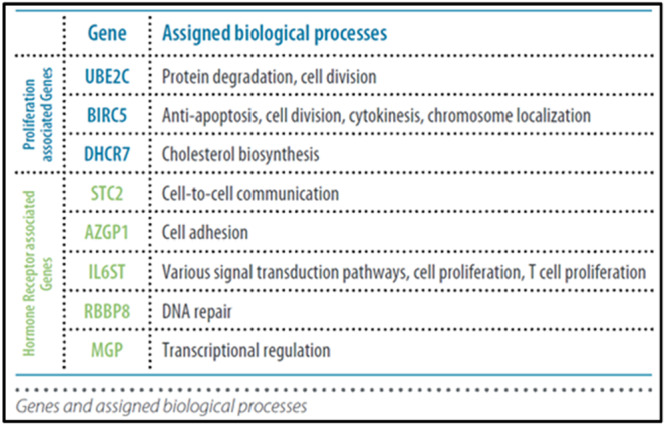
Modules and gene assessed with Endopredict test.

EndoPredict (Myriad Genetics, Salt Lake City, UT, USA) is a second‐generation RNA‐based 12‐gene expression test that determines the 10‐year absolute CT benefit for patients with ER+/HER2‐primary breast cancer as well as the risk of distant recurrence up to 15 years [27]. It can be carried out using RT‐qPCR on FFPE tumor tissue (biopsies or surgical tissues) [28]. For a precise evaluation of early and late recurrence risk, EndoPredict evaluates the activity of eight genes, including HR‐related and proliferation‐associated genes, three reference genes, and one control gene [28, 29]. The 12‐gene molecular score is combined with the clinical risk parameters of tumor size and lymph node status to determine the final EndoPredict result (EPclin Risk Score, scale 1–6). A higher probability of distant recurrence is indicated by higher EPclin scores (Figure 3) [29].

✓. **PROSIGNA (PAM‐50)**

Prosigna (Veracyte, San Francisco, CA, USA) is a second‐generation multigene expression test developed to determine distant relapse‐free survival (DRFS) 10 years following breast cancer surgery. The test is conducted on RNA isolated from FFPE breast tumor samples using the nCounter analysis system (NanoString Technologies Inc., Seattle, WA, USA). To classify surgically removed breast tumors into four intrinsic subtypes—Luminal A, Luminal B, HER2‐enriched, and basal‐like—Prosigna examines a gene signature called PAM50, which is made up of 50 genes [30, 31]. Eight housekeeping genes for normalization, six positive control genes, and eight negative control genes are also included in the PAM50 signature. The test creates a ROR score by combining data on tumor size and proliferation with information on PAM50 gene expression. Utilizing NanoString technology through the nCounter analysis system, this innovative approach bypasses the need for reverse transcription and amplification of extracted cDNA, streamlining the process and improving the accuracy of gene expression analysis. The ROR score, reported on a 0–100 scale, correlates with the likelihood of distant recurrence over a 10‐year period for postmenopausal women with HR‐positive EBC who do not receive CT [32]. For node‐negative patients, the 10‐year recurrence risk is categorized as low (scores 0–40, with a < 5% risk), intermediate (scores 41–60, with a ~ 10% risk), or high (scores 61–100, with a > 15% risk). In node‐positive patients, the recurrence risk is categorized as low (scores 0–40, with a ~ 5% risk) or high (scores 41–100, with a ~ 25% risk). The test is cleared for use in local or centralized laboratories and has received FDA 510(k) clearance in the US and the CE mark in Europe, making it suitable for FFPE tissue applications.

However, the absence of validation through prospective studies aimed at determining the clinical benefit (predictive value) of adjuvant CT across the three risk categories, limits its clinical utility [33].

✓. **BREAST CANCER INDEX**

In HR + EBC, the BCI (Biotheranostics Inc., San Diego, CA, USA) is a first‐generation RT‐PCR assay that uses the analysis of seven genes to predict whether extended ET will be beneficial and to provide a prognostic risk of late distant recurrence (the result is expressed as a percentage). A quantitative and impartial molecular evaluation of the proliferative state of tumors is produced by five cell cycle‐associated genes that go into the molecular grade index (MGI) [34, 35]. The H/I index ratio, which is provided by the final two genes (HOXB13/IL17BR), is linked to the tumor's receptiveness to ET. A centralized laboratory uses FFPE breast tumor tissue to conduct the BCI test [35].

## 4 Clinical Validity and Clinical Utility of Multigene Prognostic Test

✓. **ONCOTYPE Dx**

The pivotal TAILORx trial (NCT00310180) significantly underscored the importance of Oncotype Dx. In this study, patients with HR+/HER2− breast cancer and an Oncotype‐based RS of less than 11 exhibited favorable survival outcomes at the T1‐2, N0 stage. Additionally, for node‐negative patients with an RS between 11 and 25, ET alone was shown to be non‐inferior to chemoendocrine therapy. This finding has important implications for treatment decisions, suggesting that some patients may safely avoid CT [36]. Additionally, adjuvant treatment including taxanes and/or anthracyclines proved beneficial for both menopausal and premenopausal women with RSs of 26 or higher. Similar findings were observed in the RxPONDER trial (NCT01272037) and the phase III West German Study Group (WSG) PlanB trial (NCT01049425), both of which evaluated the efficacy of the Oncotype Dx RS in node‐positive HR+/HER2− EBC [37]. These studies reinforce the utility of Oncotype Dx in guiding treatment decisions for patients with higher RSs. The PlanB trial showed that for low‐risk patients with pN0‐1 status and an Oncotype Dx RS of 11 or less, skipping adjuvant CT resulted in impressive 5‐year disease‐free survival (DFS) rates of 94% and overall survival rates exceeding 99%. Importantly, DFS was negatively affected by high levels of Ki‐67, a marker of cell proliferation, particularly in the subgroup with RS greater than 25. However, for patients in the RS < 11 and RS 12–25 groups, Ki‐67 levels did not appear to influence prognosis [38]. The RxPONDER trial enhanced our understanding of the effectiveness of Oncotype Dx by prospectively randomizing HR+/HER2− EBC patients with 1–3 positive axillary lymph nodes and a RS of 25 or fewer to receive either ET alone or chemoendocrine therapy [37, 38]. Aligning with the findings of the PlanB trial, the results indicated that ET alone was sufficient for node‐positive postmenopausal women with RS levels of 25 or below. This reinforces the potential to avoid unnecessary CT in certain patient populations. Currently, the NRG‐BR009 trial (NCT05879926) is ongoing. This phase III study aims to determine whether adjuvant chemotherapy (ACT), in combination with ovarian function suppression (OFS) and ET, improves invasive breast cancer‐free survival (IBCFS) in premenopausal patients with HR+/HER2− EBC who have a RS between 16 and 25 for pN0 and between 0 and 25 for pN1. This is compared to treatment with OFS plus ET alone [38].

✓. **MAMMAPRINT**

Multiple studies have confirmed the prognostic and predictive value of MammaPrint, highlighting its importance in identifying patients at low ROR, thus allowing them to avoid unnecessary CT. The RASTER study, the first prospective evaluation of MammaPrint in patients with cT1‐3N0M0 breast cancer, extensively assessed the 5‐year distant‐recurrence‐free interval (DRFI) probabilities, demonstrating the clinical relevance and reliability of the assay and providing substantial validation of its prognostic accuracy [39]. Additionally, the multicenter randomized phase III MINDACT trial (NCT00433589) played a key role in demonstrating MammaPrint's clinical utility for guiding ACT decisions in EBC patients (pT1‐3, pN0‐1, pM0). The study focused on patients who were identified as having a high clinical risk through the Adjuvant! Online tool but a low genomic risk according to MammaPrint. Among these patients, those who received only ET achieved an impressive 5‐year metastasis‐free survival rate of 94.7%. This suggests that many can be spared from more aggressive treatments, regardless of their nodal status [39, 40]. Notably, early results from the MINDACT trial indicated that adding CT provided significant benefits for women under 50, increasing their 8‐year distant metastasis‐free survival from 88.6% to 93.6%. These findings emphasize the importance of using MammaPrint carefully and considering the patient's age when making treatment decisions [40].

✓. **PAM50/PROSIGNA**

PAM50 has been validated in several studies, notably in the patient populations of the ABCSG‐8 trial (NCT00291759) and the ATAC study (ISRCTN 18233230), highlighting its prognostic capabilities. These important studies showed that PAM50 effectively classifies node‐positive patients according to their recurrence risk after ET, potentially allowing low‐risk patients to avoid unnecessary CT. This highlights the utility of PAM50 in guiding treatment decisions and personalizing patient care in breast cancer [41]. In the ABCSG‐83 study, PAM50 was utilized to assess 1478 postmenopausal women with early‐stage, hormone receptor‐positive breast cancer who underwent surgery and received adjuvant hormone therapy alone. The results showed that for the ROR‐low risk group, the 10‐year survival rate free from distant recurrence was an impressive 96.7%. In the ROR‐intermediate risk group, the rate was 91.3%, while the ROR‐high risk group had a 10‐year survival rate of 79.9% [42]. These findings illustrate the prognostic value of PAM50 in stratifying patients based on their recurrence risk and guiding treatment decisions. The ROR score showed a significant correlation with the likelihood of distant recurrence when compared to common clinical factors. The study indicated that the ROR‐low risk group had a distant metastatic probability of approximately 3.5%, suggesting that CT could be safely avoided for this group due to their favorable prognosis. This reinforces the utility of the ROR score in identifying patients who may not require aggressive treatment, ultimately guiding more personalized and effective management strategies. The PROSIGNA score was linked to 10‐year distant recurrence‐free survival (DFRS) in the transATAC study, which analyzed 1007 breast cancer samples from postmenopausal patients with early‐stage, hormone‐sensitive breast cancer who underwent surgery and received only adjuvant hormonal therapy (either tamoxifen or anastrozole). The ROR score showed a statistically significant correlation with the ROR at 10 years across all patients, regardless of lymph node involvement or HER2 status. This highlights the score's value in assessing long‐term outcomes and guiding treatment decisions for this patient group [43]. The predictive value of Prosigna is currently being validated in the OPTIMA (Optimal Personalized Treatment of Early Breast Cancer using Multi‐Parameter Analysis) trial (ISRCTN42400492). This international study focuses on test‐directed CT for Luminal patients with pN0‐2 disease. In the OPTIMA trial, patients are randomly assigned to receive either ET after standard CT or ET alone if they are classified as low risk (with a Prosigna score of < 60) based on genomic analysis. This validation aims to confirm Prosigna's therapeutic effectiveness and predictive capabilities, specifically to guide treatment decisions for luminal breast cancer. By assessing outcomes based on Prosigna scores, the trial seeks to improve personalized treatment strategies for patients with early‐stage hormone receptor‐positive breast cancer [43, 44].

✓. **ENDOPREDICT**

The prognostic role of the EPclin score was evaluated in over 1500 patients from the ABCSG‐6 and ABCSG‐8 studies, demonstrating its independence as a prognostic factor when compared to clinical‐pathological factors alone. EndoPredict‘s predictive value has been extensively validated in clinical trials, including the GEICAM 9906 trial, which focused on HR+/HER2− EBC patients who were node‐positive and receiving CT [45]. However, while EndoPredict has proven to be a valuable predictive tool, it did not exhibit prognostic value regarding responses to different CT regimens. This highlights its role in guiding treatment decisions rather than predicting outcomes based on specific CT types [46].

✓. **BREAST CANCER INDEX.**

A prospective‐retrospective translational analysis of HR+ EBC patients from the IDEAL trial (BOOG 2006‐05) investigated the predictive capabilities of the BCI assay, leading to its clinical validation. The findings revealed that BCI classification significantly influenced outcomes in a randomized trial involving letrozole, administered for either 5 or 2.5 years. Patients with a high HOXB13/IL17BR (H/I) ratio experienced substantial benefits from extended ET of at least 5 years, resulting in a notable 58% and 66% reduction in the relative ROR for both the primary adjuvant treatment subgroup and the overall cohort receiving aromatase inhibitors. Conversely, patients with a low H/I ratio did not see similar advantages, highlighting the importance of the H/I ratio in guiding treatment decisions [47].

## 5 Current Guidelines on Gene Profiling Assays

According to the 2019 St. Gallen Consensus guidelines, prognostic genomic testing should be employed to guide adjuvant CT options for ER+/HER2− Stage I–II (T1/T2) node‐negative tumors, T3 node‐negative tumors, and Tx N1 cancers. The ESMO guidelines for EBC support the use of validated gene expression studies to complement clinical‐pathological data with additional prognostic and predictive insights, facilitating the assessment of the necessity for ACT (ESCAT level of evidence IA) [48]. Similarly, the NCCN guidelines recognize prognostic multigene tests (TMPs) as valuable tools for informing adjuvant systemic treatment decisions in patients with ER+/HER2− breast cancer, based on their clinical‐pathological characteristics. These guidelines underscore the importance of integrating genomic data into clinical practice to optimize treatment strategies [49]. MPTs should be used in patients with Stage I–IIA (T1N0‐1, T2N0, T2N1) ER+/HER2− breast cancer, according to the 2019 ASCO guidelines.

For individuals with negative lymph nodes, Oncotype Dx is specifically advised to distinguish between the following groups:

– For patients over 50 and RS ≤ 26 and those under 50 and RS ≤ 16, the benefits of CT are negligible or none; consequently, ET is the only recommended course of action (strong recommendation).
– For patients ≤ 50 years and RS 16–25, ACT and endocrine treatment are recommended (moderately recommended).
– RS ≥ 30: recommended for ACT, irrespective of age.
– RS 26–30: The ASCO expert panel recommends administering this medication despite the lack of specific evidence for this patient group. The only test approved by the NCCN guidelines to assess the benefits of additional CT is the Oncotype Dx test. In contrast, based on MINDACT research data, MammaPrint is recommended as a Category 1 option for patients with high clinical risk, where low genetic risk findings may justify skipping treatment.

It is recommended to use only one test per patient and tumor, as these tests may yield different results and have not been directly compared in prospective studies. The access criteria for these tests include patients with early‐stage, endocrine‐responsive breast cancer who are classified as intermediate risk and for whom the physician may evaluate the need for adjuvant treatment.

The following categories are excluded from free access to the assay: (i) Low‐risk patients, for whom only hormone therapy is recommended, (ii) high‐risk patients, for whom a combination of hormone therapy and CT is planned, (iii) patients with hormone‐positive/HER2‐negative cancer whose treatment choice (hormone therapy + CT or hormone therapy alone) is clear based on standard clinical‐pathological factors, (iv) patients with triple‐negative breast cancer (TNBC) or HER2‐positive cancer [50, 51].

Currently, neither the ASCO guidelines nor the European Society for Medical Oncology (ESMO) endorses any specific genetic test over others. Instead, they emphasize the overall value of multiple tests, particularly in complex clinical situations where the optimal choice of adjuvant therapy is unclear, such as in Luminal B patients with 1–3 positive axillary nodes. Importantly, the guidelines do not currently include explicit recommendations for the use of genetic tests for patients with four or more positive nodes. These recommendations reflect the evolving landscape of genomic testing for breast cancer and aim to assist healthcare providers in making informed treatment decisions, particularly in cases where specific guidance may be lacking [51, 52].

## 6 Comparison Among Multigene Prognostic Tests

The emergence of multiple tests for the same purpose raises significant concerns about the consistency of risk assignments when more than one test is applied to the same specimen. Comparative studies have shown that discordant risk predictions often arise when different prognostic assays are used for the same case [53]. This variability can complicate treatment decisions and introduce uncertainty in patient management. It highlights the importance of careful interpretation of test results and the need to consider the clinical context when utilizing multiple genomic tests. For instance, when six genomic signatures, including PAM50/ROR, MammaPrint, and Oncotype DX, were evaluated on the same patient cohort, each showed significant prognostic value, but the individual risk assignments often varied [54].

In a study comparing EndoPredict and Oncotype DX results in hormone receptor‐positive invasive breast cancer, there was a moderate concordance of 76% and a correlation of 0.65 between the RS and the EndoPredict (EP) Score. These differences can be attributed to the distinct biological factors emphasized by each test. Further research is necessary to explore the clinical implications of these discrepant results and how they relate to patient outcomes [55].

In another comparative study evaluating the PAM50 ROR Score alongside Oncotype DX and IHC4, the authors concluded that the ROR Score provides more prognostic information for endocrine‐treated patients with ER‐positive, node‐negative disease than the RS. They found that the ROR Score is more effective at distinguishing between intermediate‐ and higher‐risk groups, suggesting its enhanced utility in risk stratification for this patient population [56]. Recent analyses have highlighted the discrepancies between IHC‐based subtypes and intrinsic molecular subtypes. In a study evaluating PAM50 alongside IHC‐based subtypes, the discordance rate for HR+ patients was found to be 45.1%, while for HER2+ patients, it was 28.6%, according to research by Hequet et al. [57]. These findings suggest that discordance between intrinsic and IHC‐based subtypes could lead to inadequate treatment and worse survival outcomes for breast cancer patients. As a result, there may be a need to update treatment guidelines to ensure accurate identification of intrinsic subtypes, which could enhance patient management and improve outcomes [57].

Dowsett and colleagues compared the RS from the Oncotype test with the ROR score from Prosigna in a cohort of 1017 patients from the transATAC study. They found a low correlation between the RS and ROR, with a coefficient of only 0.39 [56, 57]. Additionally, 42% of patients were assigned to discordant risk groups (low, intermediate, high), with notable findings that 15% of patients classified as high risk by the ROR were classified as low risk by the Oncotype results. These findings are consistent with a recent study by Buus et al. [55], where they used the RT‐PCR version of the PAM50 assay on a cohort of 304 sequentially treated breast cancer patients. The study found that while there was generally good agreement between the PAM50 ROR score and the Oncotype DX results, PAM50 tended to assign a greater number of patients to the low‐risK category compared to Oncotype DX [55]. This suggests that PAM50 may be more conservative in its risk stratification. Importantly, in this study, approximately half of the patients classified as intermediate‐risk by Oncotype DX were assigned to the low‐risk luminal A category by PAM50.

This discrepancy in risk stratification could have important implications for treatment decisions, as some patients who might have been considered for additional CT based on their Oncotype DX classification could be identified as low‐risk by PAM50, potentially avoiding unnecessary treatment and its associated toxicities [55, 58]. Such findings highlight the need to carefully consider the specific genomic test being used, as the methods of risk stratification and the categorization of patients can vary between assays, affecting the resulting treatment recommendations. Interpreting the disparate risk estimations found across various tests necessitates an understanding of the molecular drivers and how they differ among the assays. Research indicates that, contrary to common assumptions, the RS from Oncotype Dx is more heavily influenced by factors related to the estrogen‐associated pathway. In contrast, the ROR score from Prosigna is predominantly shaped by characteristics linked to the proliferation pathway [55, 58, 59]. This highlights that the molecular features evaluated by different tumor molecular profiling tests are not interchangeable. Consequently, the results must be contextualized carefully within each specific clinical set to guide treatment decisions effectively.

## 7 Discussions

Breast cancer remains a major challenge in oncology, being the most prevalent cancer among women worldwide. Although significant progress in research and therapeutic approaches over recent decades has improved early detection and treatment, several hurdles persist. This is particularly evident in aggressive subtypes like triple‐negative breast cancer and in cases with resistance to existing therapies. Overcoming these challenges is essential to improving outcomes and survival rates for all patients affected by this disease [59].

In contemporary clinical practice, prognosis and treatment decisions for breast cancer are informed by clinical‐pathological evaluations alongside the expression of validated biomarkers, including HER2, ER, progesterone receptor (PgR), and Ki67, which indicates cell proliferation. The primary method for assessing these markers is immunohistochemistry (IHC), a technique that enables semi‐quantitative evaluation of protein expression levels on histological slides. However, inter‐ and intra‐observer variability in IHC can lead to significant discrepancies, with some studies reporting differences of up to 20%. This variability is especially critical for Ki67, which is essential in differentiating between luminal A and B subtypes of breast cancer. Such distinctions directly influence treatment decisions, including the consideration of adding cytotoxic CT to ET. Thus, ensuring consistency in IHC assessments is vital for optimizing patient care [60]. Molecular classification has greatly advanced breast cancer treatment by allowing for patient stratification based on the genetic and biological characteristics of their tumors. However, ongoing research and clinical trials remain vital to refining therapeutic strategies and personalizing treatment according to each tumor's molecular profile. This approach seeks to improve patient outcomes and address challenges such as treatment resistance. In recent years, GEP using microarray technology has provided deeper insights into the molecular complexity of breast cancer, leading to the identification of five intrinsic molecular subtypes: Luminal A, Luminal B, HER2‐enriched, Basal‐like, and normal‐like. These discoveries have been pivotal in the development of more targeted and effective treatment options [61]. Additionally, gene expression analysis has paved the way for the development of genomic tests for breast cancer, commonly known as Multigene Prognostic Tests (TMP). These tests offer critical insights into prognosis and aid in making informed treatment decisions, allowing therapies to be tailored to individual patient profiles. Retrospective studies have highlighted the effectiveness of certain assays in improving disease control and survival rates; they are are instrumental in assessing the risk of long‐term recurrence and in identifying the most appropriate treatment options. The central goal of all Multigene Prognostic Tests (TMPs) in breast cancer is to tailor therapy to the individual, ensuring that patients who need and will benefit from CT receive it, while avoiding unnecessary treatments. This approach helps to mitigate the risks of both under‐treatment and overtreatment.

Currently, five approved tests are commercially available: Oncotype DX (Genomic Health Inc., Redwood City, CA), MammaPrint (Agendia BV, Amsterdam, the Netherlands), Prosigna (PAM50; NanoString Technologies Inc, Seattle, WA), EndoPredict (Myriad Genetics Inc, Salt Lake City, Utah), and BCI [53]. While these tests have undergone strong retrospective evaluations, only Oncotype DX and MammaPrint have available data from prospective validation studies and are the most widely recognized [53, 61]. However, Prosigna distinguishes itself by not only evaluating the 10‐year ROR but also providing molecular subtyping of breast cancer based on the PAM50 signature—a panel of 50 genes developed from the foundational research by Perou, Sorlie, and their team. For patients not eligible for ACT due to comorbidities, advanced tumor stage, or high risk, or for those who clearly need CT, routine genomic testing may not be necessary. However, these tests are especially valuable in the “gray zone,” which includes tumors measuring between 1 and 3 cm with 0–3 positive lymph nodes and an intermediate proliferation index. In these cases, genomic testing can help clarify treatment decisions and identify which patients may benefit from CT, thereby personalizing therapy based on individual risk assessments [53, 62].

In a study involving patients with hormone receptor‐positive, HER2‐negative, EBC, Poulet and colleagues compared the RS from the Oncotype Dx test with MammaPrint results. They found substantial differences in risk assessment between the two tests. These discrepancies in classification are likely due to the distinct development and validation processes of each assay. This underscores the importance of understanding each test's design and the molecular factors they prioritize, as these elements can significantly influence treatment decisions for patients [62].

In a study by Dowsett and colleagues, the RS from the Oncotype test was compared with the ROR score from Prosigna in 1017 patients from the transATAC study; they found a low correlation of only 0.39 between the two scores, with 42% of patients assigned to discordant risk groups (low, intermediate, high). This highlights the variability in risk assessment provided by different genomic tests, emphasizing the need for careful interpretation of results in clinical decision‐making [55, 56].

A third study compared the RS from the Oncotype test with RT‐qPCR analysis of the PAM50 panel. The results revealed a wide distribution of RS in both Luminal A and Luminal B subtypes. Notably, 19% of Luminal B patients exhibited a low RS, with some even having scores lower than those typically reported for Luminal A patients [63].

The OPTIMA 41 trial compared various tests for stratifying recurrence risk and classifying molecular subtypes, including Prosigna, MammaPrint, MammaTyper, NexCourse Breast (IH4‐AQUA), and IHC4. This study involved 313 patients with ER+/HER2− negative breast cancer, who had either positive [1, 2, 3, 4, 5, 6, 7, 8, 64] or negative lymph nodes and tumors measuring 3 cm or larger. Patients were assigned to receive standard therapy (CT followed by adjuvant hormone therapy) or adjuvant therapy based on Oncotype DX test results. The findings revealed low concordance between the different tests for both low/intermediate‐risk and high‐risk patient groups, highlighting the variability in risk assessment among the different molecular profiling tools. This inconsistency may complicate treatment decisions and under)scores the importance of carefully interpreting test results in the context of individual patient characteristics [65].

Interpreting the disparate risk estimations identified in various studies necessitates a clear understanding of the molecular drivers associated with each test and how they differ. The discrepancies arise because, contrary to common assumptions, the RS is more influenced by factors related to the estrogen pathway, while the ROR score places greater emphasis on the proliferation pathway. This distinction highlights that the characteristics assessed by different tumor molecular profiling tests (TMPs) are not interchangeable. Therefore, it is crucial to contextualize the results based on the specific clinical scenario to make informed treatment decisions [43, 63, 65, 66]. Three approaches have been mainly examined in the scientific context, each with unique traits and restrictions: the semi‐quantitative assessment of the protein expression of important markers in breast cancer, such as HER2, Ki67, progesterone receptor (PgR), and ER, is made possible by the technique known as IHC, or IHC. The mRNA expression of the above listed important markers is quantitatively evaluated using a molecular technique called RT‐qPCR (Real‐Time Quantitative Polymerase Chain Reaction). A limitation of this method is that gene expression does not always align with protein or antigen expression. Multigene prognostic tests analyze the expression of multiple genes involved in various cellular pathways to characterize breast cancer. Importantly, these tests go beyond traditional markers like MKI67 in the proliferation pathway, highlighting the proliferation index's critical role in treatment decisions. Integrating these additional genes offers the potential for a more comprehensive understanding of the tumor's molecular profile and biological characteristics, thereby enabling more informed therapeutic choices [67].

## 8 Conclusions

In conclusion, breast cancer represents an extremely heterogeneous pathology, and thanks to significant progress in research and therapeutic strategies, it continues to have a significant social and health impact and requires a multidisciplinary, personalized approach based on the molecular and biological characteristics of the tumor. The personalization of treatment and the ability to select patients who will benefit from CT is a common goal of all gene profiling tests in breast cancer. The primary purpose of these molecular tests is to assess the necessity of ACT, helping to prevent both under‐treatment and overtreatment. Personalization of the therapeutic process is the common goal of gene profiling tests in breast cancer. Despite the differences between them, they are valid tools in the decision‐making process for planning therapy, but must be used at the discretion of the clinician and not represent the only factor in choosing treatment. It is essential to integrate them with traditional clinical‐pathological parameters to ensure an adequate therapeutic plan for the patients. Therefore, interdisciplinary collaboration and the use of new technologies represent the key to further improving the healing prospects and quality of life of patients suffering from this pathology. Advances in early diagnosis, achieved thanks to the increasingly widespread use of mammography and advanced imaging technologies, as well as the evolution of personalized therapies, including surgical, radiotherapy, CT treatments and the use of biological drugs, have contributed to a reduction in mortality and an increase in long‐term survival. However, numerous challenges still remain, especially regarding the management of the most aggressive forms, such as triple‐negative carcinoma, the prevention of relapses and the management of resistance to therapies.

## Author Contributions

Study concept and design: Francesca Collina and Marianna Rita Brogna. Data collection: Michele Del Sesto, Angela Montone, and Mariarosaria Schiano. Drafting and revision of paper: Francesca Collina, Marianna Rita Brogna, and Gerardo Ferrara. All authors have read, edited, and contributed to the content of this manuscript. This work has not been previously published and has not been considered for publication elsewhere.

## Conflicts of Interest

The authors declare no conflicts of interest.

## Data Availability

All data generated or analyzed during this study are included in this manuscript.
